# Phosphatidate phosphatase Pah1 contains a novel RP domain that regulates its phosphorylation and function in yeast lipid synthesis

**DOI:** 10.1016/j.jbc.2023.105025

**Published:** 2023-07-07

**Authors:** Geordan J. Stukey, Gil-Soo Han, George M. Carman

**Affiliations:** Department of Food Science and the Rutgers Center for Lipid Research, New Jersey Institute for Food, Nutrition, and Health, Rutgers University, New Brunswick, New Jersey, USA

**Keywords:** lipid, phospholipid, phosphatidate, diacylglycerol, triacylglycerol, Pah1, PA phosphatase, phosphorylation, RP domain, yeast

## Abstract

The *Saccharomyces cerevisiae PAH1*-encoded phosphatidate (PA) phosphatase, which catalyzes the Mg^2+^-dependent dephosphorylation of PA to produce diacylglycerol, is one of the most highly regulated enzymes in lipid metabolism. The enzyme controls whether cells utilize PA to produce membrane phospholipids or the major storage lipid triacylglycerol. PA levels, which are regulated by the enzyme reaction, also control the expression of UAS_INO-_containing phospholipid synthesis genes *via* the Henry (Opi1/Ino2-Ino4) regulatory circuit. Pah1 function is largely controlled by its cellular location, which is mediated by phosphorylation and dephosphorylation. Multiple phosphorylations sequester Pah1 in the cytosol and protect it from 20S proteasome-mediated degradation. The endoplasmic reticulum-associated Nem1-Spo7 phosphatase complex recruits and dephosphorylates Pah1 allowing the enzyme to associate with and dephosphorylate its membrane-bound substrate PA. Pah1 contains domains/regions that include the N-LIP and haloacid dehalogenase-like catalytic domains, N-terminal amphipathic helix for membrane binding, C-terminal acidic tail for Nem1-Spo7 interaction, and a conserved tryptophan within the WRDPLVDID domain required for enzyme function. Through bioinformatics, molecular genetics, and biochemical approaches, we identified a novel RP (*r*egulation of *p*hosphorylation) domain that regulates the phosphorylation state of Pah1. We showed that the ΔRP mutation results in a 57% reduction in the endogenous phosphorylation of the enzyme (primarily at Ser-511, Ser-602, and Ser-773/Ser-774), an increase in membrane association and PA phosphatase activity, but reduced cellular abundance. This work not only identifies a novel regulatory domain within Pah1 but emphasizes the importance of the phosphorylation-based regulation of Pah1 abundance, location, and function in yeast lipid synthesis.

The *Saccharomyces cerevisiae PAH1*-encoded phosphatidic acid (PA) phosphatase (PAP) is one of the most highly regulated enzymes in lipid metabolism (1, 2, 3). Pah1 catalyzes the Mg^2+^-dependent dephosphorylation of PA to produce diacylglycerol (DAG) (4, 5) (Fig. 1). The enzyme reaction controls whether cells utilize PA to produce membrane phospholipids *via* the liponucleotide intermediate CDP-DAG or the major storage lipid triacylglycerol (TAG) *via* the intermediate DAG (1, 2, 3) (Fig. 1). The expression of *PAH1* is repressed during exponential phase (6) when cells are actively dividing and PA is needed to synthesize membrane phospholipids (7, 8). As cells progress into the stationary phase, enzyme expression is derepressed (6) as energy storage takes priority over cell division and PA is increasingly used for the synthesis of TAG (7, 8). PA levels, which are mediated by the PAP reaction, also control the transcriptional regulation of UAS_INO__-_containing phospholipid synthesis genes *via* the Henry (Opi1/Ino2-Ino4) regulatory circuit (2, 8, 9, 10, 11, 12).

**Figure 1:**
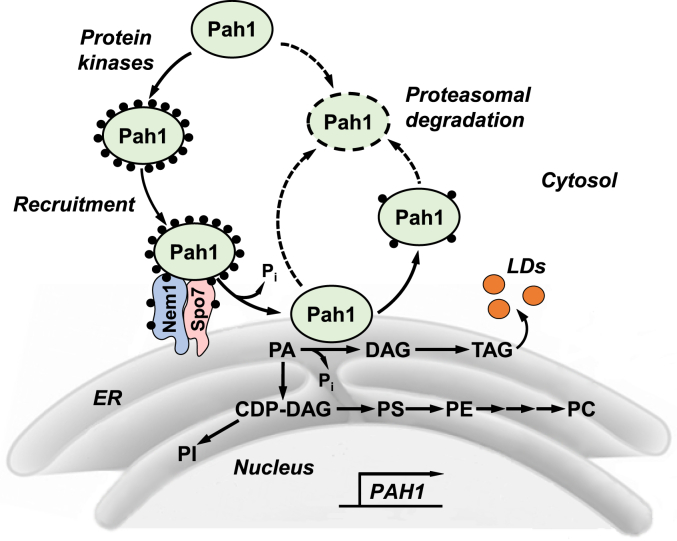
**Model of the phosphorylation/dephosphorylation-mediated regulation of Pah1 PAP.** Following its expression, which is regulated by the growth phase and nutrients, Pah1 is susceptible to proteolytic digestion. The enzyme is stabilized by its phosphorylation but sequestered in the cytosol apart from its membrane-associated substrate PA. Pah1 translocates to the nuclear/ER membrane through the Nem1-Spo7 phosphatase complex, which dephosphorylates Pah1 allowing for its interaction with the membrane surface. Dephosphorylated Pah1 catalyzes the dephosphorylation of PA to form DAG, which is subsequently acylated to form the TAG that is stored in cytoplasmic lipid droplets (*LD*). When the PAP activity is attenuated, the substrate PA is more channeled into membrane phospholipids (*e.g.*, phosphatidylserine (*PS*), phosphatidylethanolamine (*PE*), phosphatidylcholine (*PC*), and phosphatidylinositol (*PI*)) *via* CDP-DAG. Unphosphorylated Pah1 or Pah1 phosphorylated by protein kinase C dissociates from the membrane and is subject to proteasome-mediated degradation. Detailed aspects of this model are reviewed elsewhere (2, 46).

WT cells synthesize the major phospholipids phosphatidylcholine and phosphatidylethanolamine from PA *via* the CDP-DAG pathway, whereas mutants defective in this pathway (13, 14, 15, 16, 17, 18, 19, 20) should synthesize these phospholipids from DAG produced by the PAP reaction *via* the CDP-choline (21, 22, 23, 24) and CDP-ethanolamine (25, 26, 27, 28) branches of the Kennedy pathway (8, 12, 29, 30). The physiological importance of the enzyme is highlighted by numerous studies that examine effects of the *pah1*Δ mutation on lipid synthesis and cell physiology; loss of PAP activity gives rise to a diverse set of deleterious phenotypes (5, 31, 32, 33, 34, 35, 36, 37, 38, 39, 40, 41, 42, 43) that ultimately result in a shortened chronological life span and apoptotic cell death in the stationary phase (reviewed in ref. (2)).

Pah1 is a peripheral membrane protein and its PAP activity occurs at the nuclear/ER membrane surface (5, 44, 45) (Fig. 1). The subcellular location of Pah1 is controlled by the posttranslational modifications of phosphorylation and dephosphorylation (46) (Fig. 1). Pah1 is phosphorylated by multiple protein kinases (47, 48, 49, 50, 51, 52, 53) (Fig. 2*A*) and, in general, the phosphorylated enzyme is localized to the cytosol (47, 54) (Fig. 1). The phosphorylation not only serves to sequester Pah1 to the cytosol, but it also protects the enzyme from degradation by the 20S proteasome (55, 56). Some phosphosites are unique to a specific protein kinase (*e.g.*, Ser-10, Ser-511, Ser-814), while others (*e.g.*, Ser-602, Ser-677, Ser-748) are common to multiple protein kinases (46) (Fig. 2*A*). Some phosphorylations are hierarchical in nature where the phosphorylation at one site affects the phosphorylation at another site (46, 53). Additionally, phosphorylations by some protein kinases stimulate (*e.g.*, casein kinase I (52)) or inhibit (*e.g.*, Pho85-Pho80 (47), Rim11 (53)) PAP activity.

**Figure 2:**
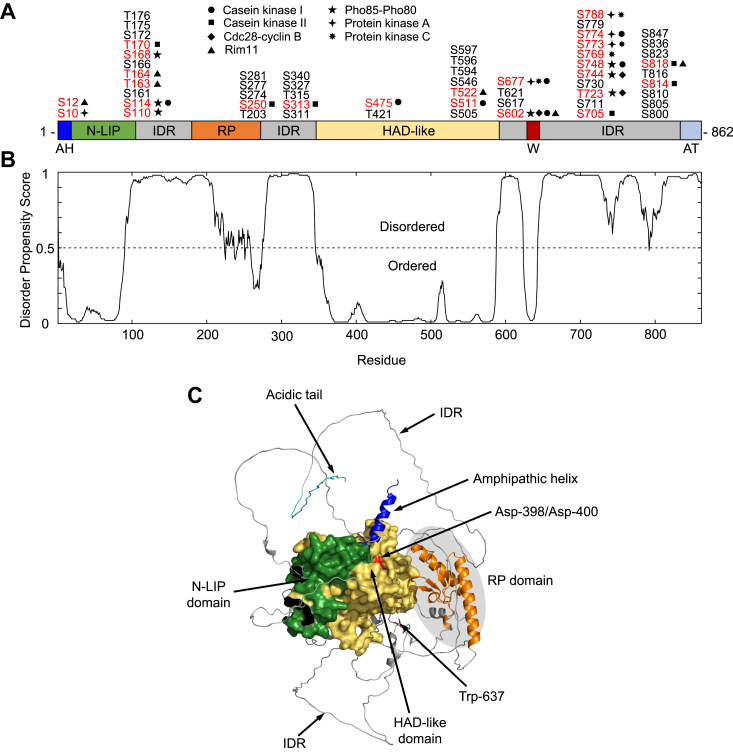
**Domains/regions, phosphosites, ordered/disordered domain predictions, and AlphaFold model of Pah1.***A*, the linear schematic displays the domains/regions of Pah1; amphipathic helix (*AH*); conserved N-LIP domain; conserved RP domain; conserved haloacid dehalogenase (*HAD*)-like domain; conserved tryptophan (*W*) residue; acidic tail (*AT*); and intrinsically disordered regions (*IDR*). The serine (*S*) and threonine (*T*) residues which have been shown to be phosphorylated are indicated at the approximate location on the schematic. The residues known to be phosphorylated by specific protein kinases (*symbols*) are indicated in *red*. *B*, the amino acid sequence of Pah1 (UniProt: P32567) was analyzed by the DISOPRED algorithm which predicts the presence of IDRs. A disorder propensity score >0.5 indicates a greater likelihood of intrinsic disorder, and a score of <0.5 indicates a greater likelihood of protein folding. *C*, predicted AlphaFold structure of Pah1 was visualized by the PyMol program. The domains/regions, catalytic Asp-398/Asp-400 residues within the HAD-like domain, Trp-637, and the RP domain are indicated.

The translocation of Pah1 from the cytosol to the nuclear/ER membrane is mediated by the Nem1 (catalytic subunit)-Spo7 (regulatory subunit) protein phosphatase complex (57) (Fig. 1). The complex, which functionally activates Pah1, is responsible for the recruitment and dephosphorylation of Pah1 (31, 45, 47, 48, 54, 57, 58, 59) that allows the enzyme to hop onto the membrane, scoot along the surface to recognize the substrate PA and catalyze its dephosphorylation (60) (Fig. 1). Moreover, the substrate PA stimulates Nem1-Spo7 phosphatase activity, and this regulatory effect is governed by the nature of the phosphate headgroup but not by the fatty acyl moiety of PA (61).

There are multiple domains/regions interspersed by intrinsically disordered regions (IDRs) throughout the primary structure of Pah1 that are important to its location, function, and regulation (Fig. 2*A*). The N-LIP and the haloacid dehalogenase (HAD)-like domains are required for PAP activity (5, 38). The HAD-like domain contains the D*X*D*X*(T/V) catalytic motif that is essential to PAP activity and Pah1 function (38). Crystal structures of the *Tetrahymena thermophila* Pah2 homolog that lacks IDRs show that the N-LIP and HAD-like domains interact to form the functional catalytic core (62). This interaction is depicted in the AlphaFold structure of *S. cerevisiae* Pah1 (Fig. 2*C*). The interaction between Pah1 and the Nem1-Spo7 complex is mediated by its C-terminal acidic tail (58), whereas its N-terminal amphipathic helix is required for the membrane interaction following its dephosphorylation by the Nem1-Spo7 complex (45). Within the IDR that constitutes most of the C-terminal half of Pah1 resides the WRDPLVDID domain that contains a conserved tryptophan residue (Trp-637) (63) that is critical to the *in vivo* function of Pah1 but is not required for its PAP activity *per se* (63, 64). Most of the phosphorylation sites, which are critical for the targeting, translocation, and regulation of Pah1 are located within the IDRs (46) (Fig. 2*A*). Also of note is that the unphosphorylated form of Pah1 is highly susceptible to 20S proteasome-mediated degradation, but its phosphorylation by multiple protein kinases at sites within the IDRs stabilizes the enzyme against the proteasomal degradation (56).

The stretch between the N-LIP and HAD-like domains of Pah1 has been thought to be a continuous IDR (63, 65). However, using bioinformatics approaches we identified a 93-amino acid sequence in the middle of the N-terminal IDR that shows far less confidence regarding the presence of its intrinsic disorder (Fig. 2*B*). Additionally, the AlphaFold model of Pah1 predicts structure for this region and it is well conserved among multiple fungal Pah1 orthologs. Taken together, these analyses led to the hypothesis that this region represents a functional domain that we named RP (for *r*egulation of *p*hosphorylation). We showed that the ΔRP mutation in Pah1 results in a 57% reduction in the endogenous phosphorylation of the enzyme, an increase in membrane association and PA phosphatase activity, and a gain of Pah1 function in the absence of the Nem1-Spo7 complex. This work not only identifies a novel regulatory domain within Pah1 but emphasizes the importance of the phosphorylation-based regulation of Pah1 abundance, location, and function in yeast lipid synthesis.

## Results

### Identification of a previously unrecognized conserved domain in Pah1

**Figure 3:**
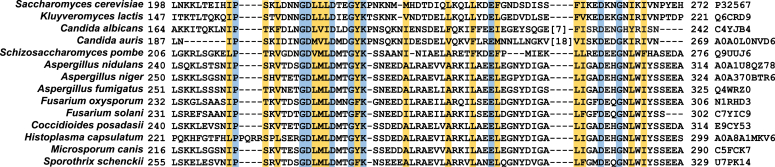
**RP domain sequence alignment of Pah1 orthologs.** Clustal Omega alignment of the RP domain in yeast species. The alignment shows nine conserved amino acid residues (*blue*) across Pah1 orthologs and 15 residues (*yellow*) for which the amino acid properties are maintained.

Large stretches of *S. cerevisiae* Pah1 contain IDRs (Fig. 2*A*) (56) precluding structural information through crystallographic and cryo-electron microscopic approaches. Accordingly, we sought to analyze Pah1 for structural information using bioinformatics. We utilized several algorithms such as DISOPRED (66), flDPnn (67), and FoldIndex (68) to analyze the presence of ordered regions and IDRs in the protein (Fig. 2*B*). Consistent with previous findings (56), DISOPRED identified ordered regions matching the residues of the N-LIP and HAD-like domains, and the WRDPLVDID domain that contains Trp-637, an essential residue for Pah1 function (63, 64) (Fig. 2*B*). These algorithms also predict a significant stretch (*i.e.*, amino acids 180–272) within the middle region of the N-terminal IDR that is not disordered (Fig. 2*B*). We refer to this region as the RP (*r*egulation of *p*hosphorylation) domain. The position of the RP domain is highlighted in the AlphaFold (69, 70) model of Pah1, which depicts the close interactions between the structured N-LIP and HAD-like catalytic domains (38, 62) (Fig. 2*C*). A BLAST (71) analysis reveals that the RP domain to be evolutionarily well conserved among fungal Pah1 orthologs, which includes nine conserved residues and 15 residues with conservation of amino acid properties such as hydrophobicity and charge (Fig. 3).

### Effects of the ΔRP mutation on cellular attributes that depend on Pah1 function

The effects of the ΔRP mutation on key cellular attributes that depend on Pah1 were examined. Deletion of the RP domain is not expected to have major deleterious effects on the Pah1 structure as its removal does not compromise the PAP activity and physiological functions of Pah1 (63). The WT and ΔRP mutant forms of the enzyme were expressed from a single-copy plasmid driven by the native *PAH1* promoter (Table 1) in *pah1*Δ and *pah1*Δ *nem1*Δ mutant cells. The single-copy plasmid was used to approximate the endogenous *PAH1* expression level, and the *nem1*Δ background was used to assess whether the effects of the ΔRP mutation on Pah1 function is dependent on its dephosphorylation by the Nem1-Spo7 complex (48, 54).

**Table 1:** Strains and plasmids used in this study

Strain or plasmid	Genotype or relevant characteristics	Source or reference
Strain		
*S. cerevisiae*		
RS453	*MAT***a***ade2-1 his3-11,15 leu2-3112 trp1-1 ura3-52*	(41)
SS1026	*pah1*Δ*::TRP1* derivative of RS453	(31)
SS1132	*pah1*Δ*::TRP1 nem1*Δ*::HIS3* derivative of RS453	(48)
*E. coli*		
DH5⍺	F^-^ Φ80 *lacZ*ΔM15Δ (*lacZYA-argF*)U169 *deoR rec*A1 *end*A1 *hsd*R17(r_k_^-^ m_k_^+^) *pho*A *sup*E44 λ^−^*thi*-1 *gyr*A96 *rel*A1	(95)
NiCo21(DE3)pLysSRARE2	*can::CBD fhuA2 [Ion] ompT gal (λ DE3) [dcm] amA::CBD sly::CBD glmS6Ala* Δ*hsdS* λ *DE3* = λ *sBamHIo* Δ*EcoRI-B int::(lacI::PlacUV5::T7 gene1) i21* Δ*nin5* pLysSRARE2	New England Biolabs
Plasmid		
pET-15b	*E. coli* expression vector with N-terminal His_6_-tag fusion	Novagen
pGH313	*PAH1* coding sequence inserted into pET-15b	(5)
pGS108	*PAH1*(Δ180–272) derivative of pGH313	This study
pRS415	Single-copy number *E. coli*/yeast shuttle vector with *LEU2*	(105)
pGH315	*PAH1* inserted into pRS415	(48)
pGH315-ΔN-NCR*b*	*PAH1*(Δ186–266) derivative of pGH315	(63)
pYES2	High-copy number *E.coli*/yeast shuttle vector with *URA3* and *GAL1* promoter	Thermo Fisher Scientific
pGH452	*PAH1*-TAP in pYES2 with calmodulin binding peptide DNA sequence removed from the TAP tag	(64)
pGS104	*PAH1*(Δ180–272) derivative of pGH452	This study
YCplac33-SEC63-GFP	*SEC63-GFP* fusion inserted into the *CEN/URA3* vector	(41)

#### TAG content

**Figure 4:**
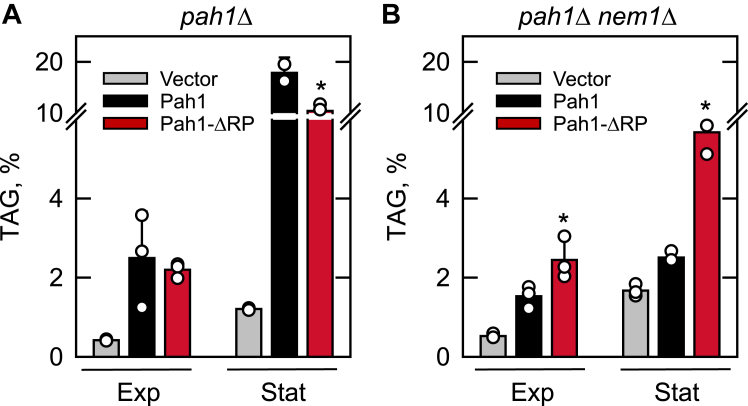
**Effect of the ΔRP mutation on TAG content in cells with and without the Nem1-Spo7 protein phosphatase complex.** The *pah1*Δ (*A*) and *pah1*Δ *nem1*Δ (*B*) cells expressing WT or ΔRP mutant forms of Pah1 from single-copy plasmids pGH315 and pGH315-ΔN-NCRb, respectively, were grown at 30 ^°^C to the exponential (*Exp*) and stationary (*Stat*) phases of growth in SC-Leu medium containing [2-^14^C]acetate (1 μCi/ml). Lipids were extracted, separated by one-dimensional TLC, and subjected to phosphorimaging and ImageQuant analysis. The percentage shown for TAG was normalized to the total ^14^C-labeled chloroform-soluble fraction. The data are the means ± SD (*error bars*) from three separate experiments. The individual data points are also shown. ∗*p* < 0.05 *versus* TAG of WT cells.

The synthesis of TAG is largely dependent on the Pah1 function (5), and accordingly, we examined the effect of the ΔRP mutation on the content of the neutral lipid in exponential and stationary phase cells. The cells expressing the WT or mutant forms of Pah1 were labeled with [2-^14^C]acetate, followed by the extraction and analysis of TAG. As described previously (5, 6), the TAG content of cells expressing WT Pah1 with the Nem1-Spo7 complex was 7-fold greater in the stationary phase when compared with the exponential phase (Fig. 4*A*). The ΔRP mutation in Pah1 did not have a major effect on the TAG content of the Nem1-Spo7 complex-containing cells in the exponential phase, but caused a decrease in TAG content in the stationary phase suggesting some dysregulation (Fig. 4*A*). As described previously (6), the *nem1*Δ mutation had a major effect on the TAG content of cells expressing WT Pah1 in both the exponential and stationary phases of growth (Fig. 4*B*). The TAG content in the exponential and stationary phases, respectively, of the cells with WT Pah1 that lack the Nem1-Spo7 complex were 1.6-fold and sevenfold lower when compared with the cells containing the Nem1-Spo7 complex. This highlights the importance of the Nem1-Spo7 complex in the recruitment, dephosphorylation, and activation of Pah1 at the nuclear/ER membrane (46). Strikingly, the ΔRP mutation complemented the negative impact of the *nem1*Δ mutation and lack of the Nem1-Spo7 complex in cells; the TAG contents of exponential and stationary phase cells expressing Pah1-ΔRP were 1.6-fold and 2.2-fold, respectively, greater when compared with the same cells expressing WT Pah1 (Fig. 4*B*).

#### Lipid droplet formation

**Figure 5:**
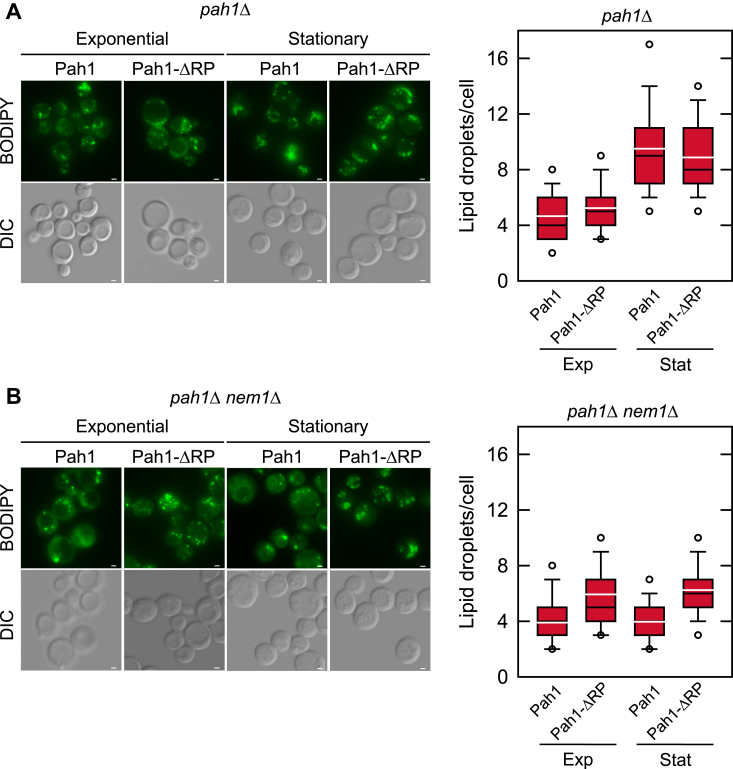
**Effect of the ΔRP mutation on lipid droplet formation in cells with and without the Nem1-Spo7 protein phosphatase complex.** The *pah1*Δ (*A*) and *pah1*Δ *nem1*Δ (*B*) cells expressing WT or ΔRP mutant forms of Pah1 from single-copy plasmids pGH315 and pGH315-ΔN-NCR*b*, respectively, were grown at 30 ^°^C in SC-Leu medium to the exponential and stationary phases of growth, and then stained with BODIPY 493/503. The stained lipid droplets were visualized by fluorescence microscopy, and the number of lipid droplets was counted from ≥300 cells (≥5 fields of view). *A* and *B left*, the images shown are representative of multiple fields of view. *White bar*, 2 μm. *A* and *B right*, the data are presented by the *box plot*. The *black* and *white lines* are the median and mean values, respectively, and the *white circles* are the outlier data points of the fifth and 95th percentile. *DIC*, differential interference contrast.

We next examined the effect of the ΔRP mutation on the formation of cytoplasmic lipid droplets. As described previously (33, 72) for the Nem1-Spo7 complex-containing cells expressing WT Pah1, the number of lipid droplets per cell was ∼2-fold greater in the stationary phase when compared with the exponential phase (Fig. 5*A*). The ΔRP mutation did not affect lipid droplet formation in cells with the Nem1-Spo7 complex. Consistent with the negative effect the *nem1*Δ mutation has on TAG content, the lipid droplet formation in the stationary phase was compromised by loss of Nem1-Spo7 complex function (72) (Fig. 5*B*). However, in cells lacking the Nem1-Spo7 complex, the ΔRP mutation had a positive effect on lipid droplet formation. In both exponential and stationary phase cells the ΔRP mutation in Pah1 resulted in a 1.5-fold increase in lipid droplet formation (Fig. 5*B*).

#### Nuclear/ER morphology

**Figure 6:**
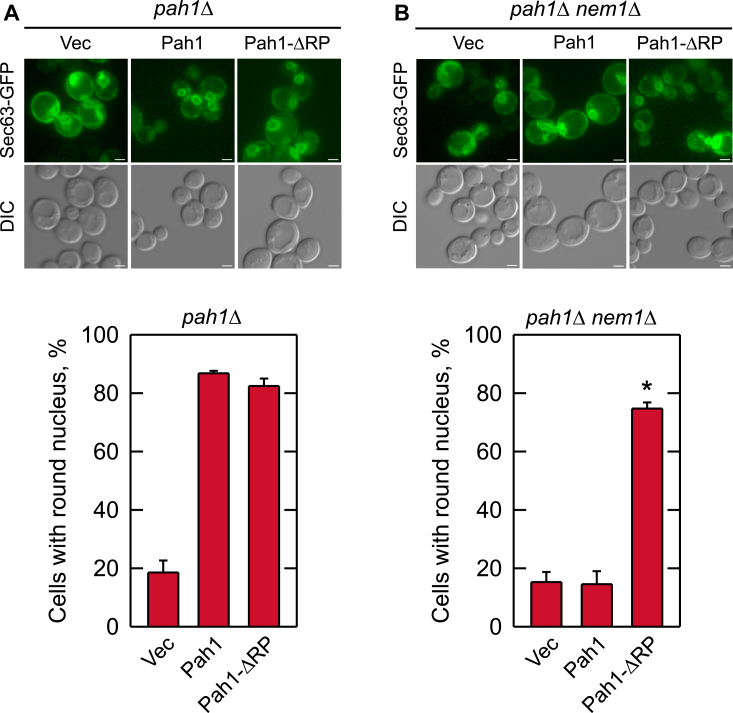
**Effect of the ΔRP mutation on nuclear/ER morphology in cells with and without the Nem1-Spo7 protein phosphatase complex.** The *pah1*Δ (*A*) and *pah1*Δ *nem1*Δ (*B*) cells expressing WT or ΔRP mutant forms of Pah1 from single-copy plasmids pGH315 and pGH315-ΔN-NCR*b*, respectively, and expressing the GFP-tagged nuclear/ER membrane marker Sec63 from plasmid YCplac33-*SEC63-GFP* were grown at 30 ^°^C in SC-Leu-Ura medium to the exponential phase of growth. *Upper*, the fluorescence signal of the Sec63-GFP was visualized by fluorescence microscopy. The images shown are representative of multiple fields of view. *White bar*, 2 μm. *Lower*, the percentage of cells with round nuclear/ER morphology was determined from ≥4 fields of views (≥200 cells). The data are averages ± S.D. (*error bars*). ∗*p* < 0.05 *versus* round nucleus of WT cells. *DIC*, differential interference contrast.

*S. cerevisiae* cells defective in any component of the Pah1/Nem1-Spo7 phosphatase axis exhibit an irregularly shaped nucleus due to the aberrant expansion of the nuclear/ER membrane (31, 38, 57, 72) (Fig. 6, *vector control*). This phenotype is attributed to loss of Pah1 PAP activity, accumulation of PA, and increased synthesis of membrane phospholipids (5, 31, 32, 38). We examined the effect of the ΔRP mutation in Pah1 on the nuclear/ER morphology in exponential phase cells by expression of the ER marker Sec63-GFP (Fig. 6). We observed that over 80% of the Nem1-Spo7 complex-containing cells expressing either WT or the ΔRP mutant form of Pah1 displayed round nuclei (Fig. 6*A*). However, as described previously (57, 72), in cells lacking the Nem1-Spo7 complex less than 20% of cells expressing WT Pah1 exhibited a round nucleus (Fig. 6*B*). In marked contrast, almost 80% of cells lacking the Nem1-Spo7 complex, but expressing the ΔRP mutant form of Pah1, showed normal round nuclei (Fig. 6*B*).

### Effect of the ΔRP mutation on the abundance and cellular distribution of Pah1

**Figure 7:**
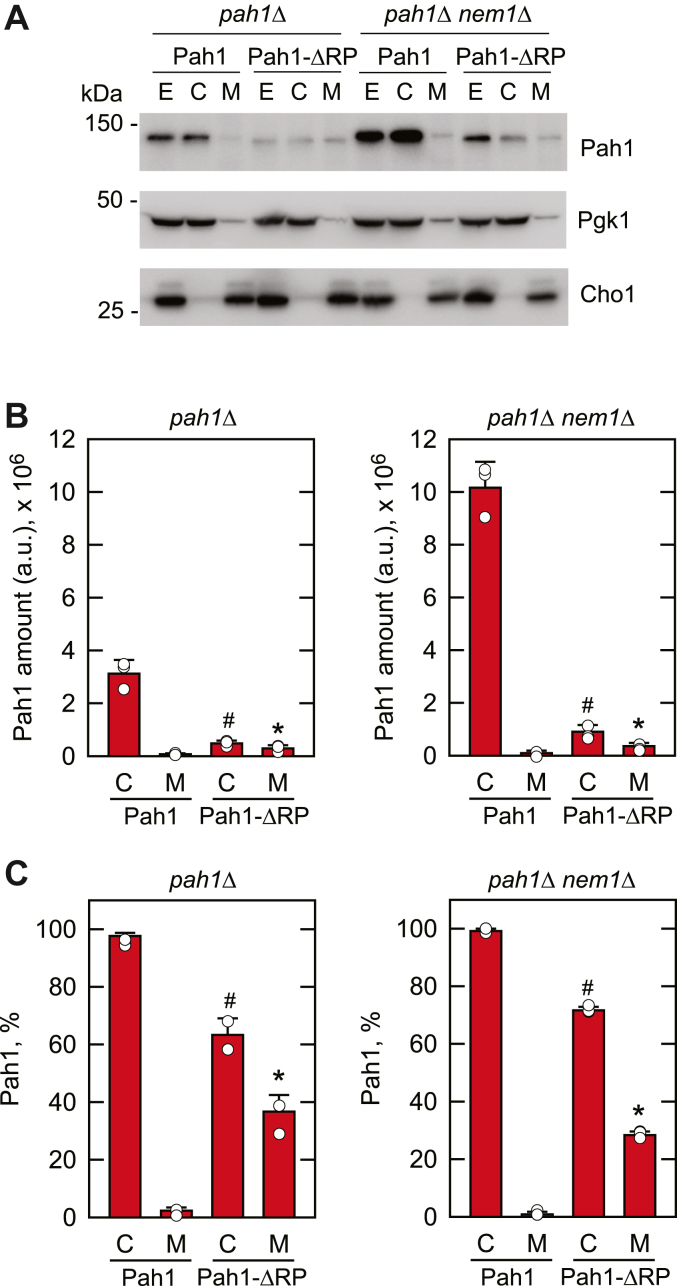
**Effect of the ΔRP mutation on the abundance and cellular localization of Pah1 in cells with and without the Nem1-Spo7 protein phosphatase complex.***A*, the *pah1*Δ (*left*) and *pah1*Δ *nem1*Δ (*right*) cells expressing WT or ΔRP mutant forms of Pah1 from single-copy plasmids pGH315 and pGH315-ΔN-NCR*b*, respectively, were grown at 30 ^°^C to the exponential phase in SC-Leu medium. Cell extracts (E) were fractionated into the cytosolic (C) and membrane (M) fractions by centrifugation at 100,000*g* for 1 h. The membrane fraction was suspended in the same volume as the cytosolic fraction of lysis buffer, and equal volumes of the fractions were subjected to SDS-PAGE using 12% polyacrylamide gels. Proteins were transferred to the PVDF membrane and probed with anti-Pah1, anti-Pgk1, and anti-Cho1 antibodies. The positions of Pah1, Pgk1 (cytosol marker), Cho1 (ER membrane marker), and molecular mass standards are indicated. The immunoblots shown are representative of three independent experiments. *B*, the immunoblots were imaged using an iBright 1500 Imager and analyzed by iBright Analysis software; the intensity in arbitrary units (*a.u.*) of the signals was determined and averaged for three experiments ± standard deviation (*error bars*). *C*, the data in panel *B* was used to calculate the relative amounts of Pah1 in the cellular fractions. The individual data points are also shown. ∗*p* < 0.05 *versus* membrane of WT cells. #*p* < 0.05 *versus* cytosol of WT cells.

The effects of the ΔRP mutation on the abundance and cellular location of Pah1 were examined. The WT and ΔRP mutant forms of the enzyme were expressed from the single-copy plasmid in the cells with (*pah1*Δ) and without (*pah1*Δ *nem1*Δ) the Nem1-Spo7 complex. Cultures were grown to the mid-exponential phase, cell extracts (*E*) were prepared and fractionated into the cytosolic (*C*) and membrane (*M*) fractions, and Pah1 was analyzed by immunoblotting with anti-Pah1 antibody (Fig. 7*A*). Exponential phase cells were used for this experiment because Pah1 is subject to proteasomal degradation in the stationary phase and the protein is difficult to visualize (55). As described previously (48), the hyperphosphorylation of WT Pah1, imparted by lack of the Nem1-Spo7 complex, was associated with greater protein abundance when compared with cells containing the protein phosphatase (Fig. 7*A*). As expected, in both genetic backgrounds, nearly all WT Pah1 was associated with the cytosolic fraction (Fig. 7). The amount of WT enzyme in the cytosolic fraction of cells lacking Nem1-Spo7 was 3.3-fold greater when compared with its amount in cells with the complex (Fig. 7*B*). Furthermore, only about 1 to 2% of the WT enzyme was associated with the membrane fraction (Fig. 7*C*). These observations confirm the importance of phosphorylation to the localization and abundance of WT Pah1 (48, 55, 56). The ΔRP mutation had a major effect on the amount of Pah1 (Fig. 7). For cells with and without the Nem1-Spo7 complex, the amounts of the cytosolic ΔRP mutant enzyme was 6.5-fold and 11-fold lower, respectively, when compared with cytosolic WT enzyme (Fig. 7*B*). The lower abundance of the ΔRP mutant protein is not expected to be due to a defect in gene expression since all genetic constructs were expressed from the same plasmid driven by the native *PAH1* promoter, but instead due to increased sensitivity to 20S proteasomal degradation. The ΔRP mutation also had a major effect on the cellular distribution of Pah1 (Fig. 7). Despite its lower total abundance, more ΔRP mutant Pah1 was associated with the membrane fraction when compared with the WT protein; this was observed in cells with (3.9-fold) and without (4-fold) the Nem1-Spo7 complex (Fig. 7*B*). The relative amounts of the ΔRP mutant protein associated with the membrane for cells with and without Nem1-Spo7 were 37% and 28%, respectively (Fig. 7*C*).

### Effect of the ΔRP mutation on the PAP activity of Pah1

**Figure 8:**
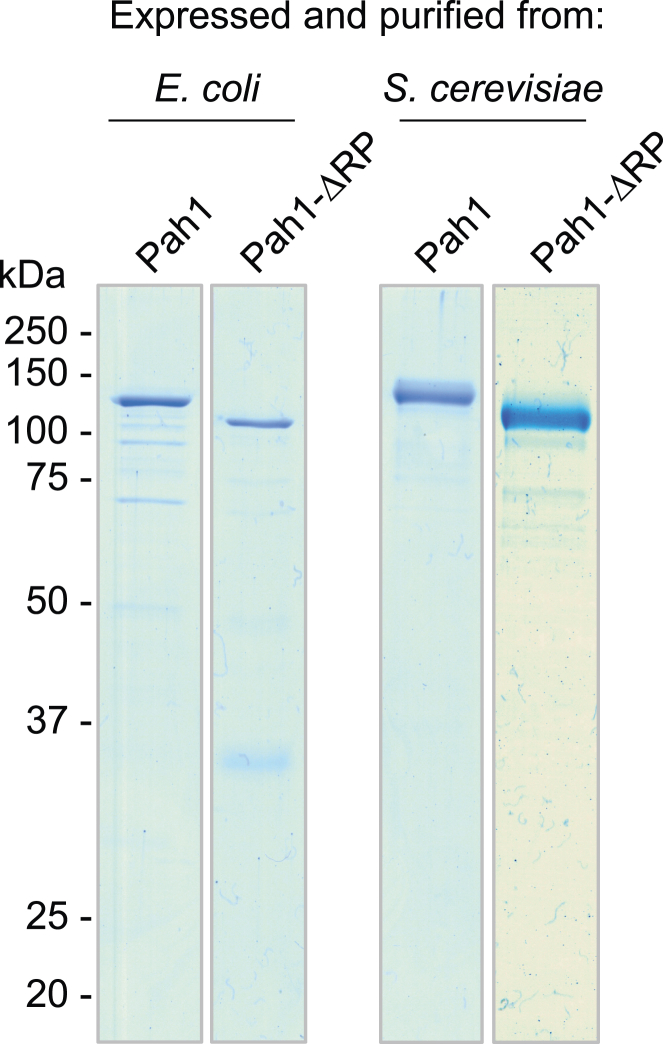
**Purification of the WT and ΔRP mutant forms of Pah1.** The WT and ΔRP mutant forms of Pah1 were expressed and purified from *E. coli* and *S. cerevisiae* (*i.e.*, *pah1*Δ *nem1*Δ mutant cells lacking the Nem1-Spo7 complex). The purified enzymes (0.5 μg) were subjected to SDS-PAGE analysis using 12% polyacrylamide gels. The positions of Pah1 and molecular mass standards are indicated.

**Figure 9:**
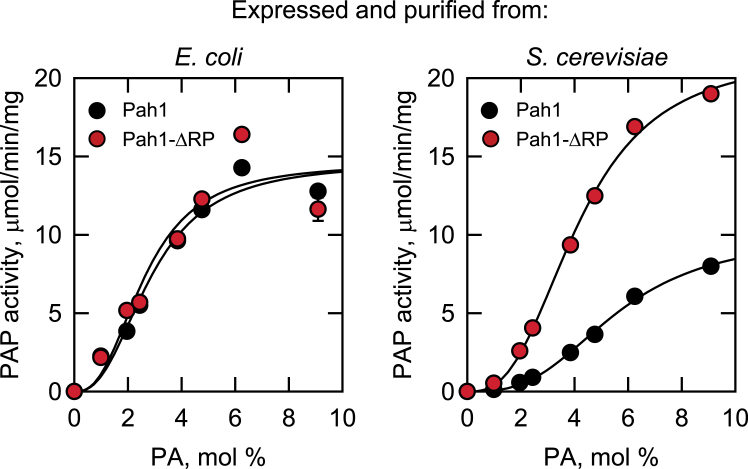
**Effect of the ΔRP mutation on the PAP activity of Pah1 expressed and purified from *E. coli* and *S. cerevisiae*.** WT and ΔRP mutant forms of Pah1 purified from *E. coli* (*unphosphorylated*) and *S. cerevisiae* (*phosphorylated*) were assayed for PAP activity by monitoring the release of water-soluble ^32^P_i_ from chloroform soluble [^32^P]PA. The molar concentration of PA was maintained at 0.2 mM while adjusting the surface concentration of PA (mol %) by varying the molar concentration of Triton X-100 (74). The values are the average of three separate experiments ± SD (*error bars*). Some of the error bars are hidden behind the *circles*.

We questioned whether deletion of the RP domain has an effect on the innate PAP activity of Pah1. To test this in a well-defined system, the WT and ΔRP forms of Pah1 were expressed and purified to near homogeneity from *Escherichia coli* and *S. cerevisiae* (Fig. 8). Pah1 heterologously expressed in *E. coli* is not subject to endogenous phosphorylation, whereas the enzyme expressed in *S. cerevisiae* is endogenously phosphorylated by multiple protein kinases (5, 46, 48, 54) (Fig. 2*A*). Preserving the hyperphosphorylated state of the enzymes was ensured by expressing them in cells (*i.e.*, *pah1*Δ *nem1*Δ mutant) lacking the Nem1-Spo7 protein phosphatase complex (54). The PAP activity of the purified enzyme preparations was measured with respect to the surface concentration of PA using the well-characterized Triton X-100/PA-mixed micelle system (5, 73, 74). The kinetic properties that include *k*_cat_, *K*_m_ and Hill number are summarized in Table 2. As described previously (5), the PAP activity exhibited by the *E. coli*-expressed unphosphorylated form of Pah1 displayed positive cooperative kinetics with respect to PA (Fig. 9, *left*). The ΔRP mutation did not have a significant effect on the kinetic properties of the unphosphorylated form of the enzyme (Fig. 9, *left*, Table 2). These data indicated that the RP domain has no effect on the catalytic competency of Pah1. We then questioned whether the phosphorylation state of Pah1 in conjunction with the ΔRP mutation would affect the kinetic properties of PAP activity. Accordingly, the kinetic analysis was performed on the WT and ΔRP forms of the enzymes expressed and purified from *S. cerevisiae*. As described previously (47, 54), the phosphorylation state of WT Pah1 had major effects on the kinetic properties of PAP activity (Fig. 9, *right*, Table 2). The *k*_cat_ and *K*_m_ values of the phosphorylated WT enzyme were ∼1.5-fold lower and ∼2-fold greater, respectively, when compared with the *E. coli*-expressed unphosphorylated WT enzyme. Consequently, the specificity constant (*k*_cat_/*K*_m_) for the PAP activity of phosphorylated WT Pah1 was ∼3-fold lower when compared with the unphosphorylated form of the WT enzyme (Table 2). The ΔRP mutation had a major effect on the PAP activity of the Pah1 expressed and purified from *S. cerevisiae* (Fig. 9, *right*, Table 2). It caused ∼2-fold increase in *k*_cat_ and a 1.3-fold decrease in the *K*_m_ for PA when compared with the WT enzyme expressed in *S. cerevisiae*; the specificity constant for PAP activity of the ΔRP mutant enzyme was 2.6-fold greater when compared with the WT enzyme. The kinetics of the ΔRP mutant enzyme expressed and isolated from *S. cerevisiae* were more similar to the unphosphorylated WT and ΔRP mutant forms expressed and purified from *E. coli* (Fig. 9, Table 2). The Hill number for PA of the WT and ΔRP mutant forms of Pah1 was not majorly affected by the phosphorylation state of the enzymes (Table 2).

**Table 2:** Kinetic constants for Pah1 and Pah1-ΔRP

Pah1	*k*_cat_	*K*_*m*_	*k*_cat_/*K*_*m*_	Hill no.
	*s*^*−1*^	*mol %*	*mol %*^*−1*^*s*^*−1*^	*n*
Unphosphorylated				
Pah1	23.1	2.8	8.2	2.5
Pah1-ΔRP	20.4	2.6	7.8	2.5
Phosphorylated				
Pah1	15.7	5.5	2.8	2.9
Pah1-ΔRP	30.1	4.1	7.3	2.8

Data were calculated from the plots shown in Figure 9.

### Effect of the ΔRP mutation on the endogenous phosphorylation of Pah1

**Figure 10:**
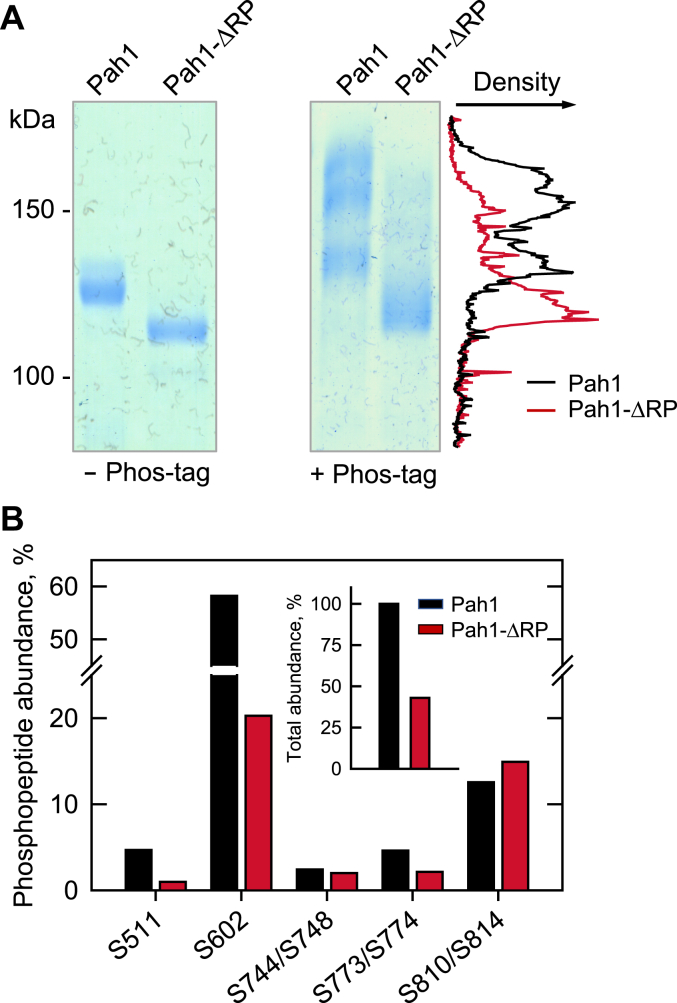
**Effect of the ΔRP mutation on the endogenous phosphorylation of Pah1.** The WT and ΔRP mutant forms of Pah1 were expressed and purified from *S. cerevisiae*. *A*, the endogenously phosphorylated purified enzymes were subjected to SDS-PAGE analysis using 5% polyacrylamide gels in the absence (*left*) and presence (*right*) of 20 μM Phos-tag and 100 μM MnCl_2_. The amounts of Pah1 applied to the polyacrylamide gels without and with Phos-tag were 0.5 and 1.5 μg, respectively. The resolved proteins were stained with *Coomassie blue*. The positions of molecular mass standards are indicated for the polyacrylamide gel lacking Phos-tag (*left*). The signal intensities of the WT and ΔRP mutant forms of Pah1 along their migration in the polyacrylamide gel containing the Phos-tag reagent were measured using the line graph function of ImageQuant software. The overlay of the densitograms of the WT and ΔRP mutant forms of Pah1 is shown on the *right side* of the Phos-tag gel. The data are representative of duplicate experiments. *B*, the WT and ΔRP forms of Pah1 in SDS-polyacrylamide gel (12%) slices were extracted, reduced, alkylated, and digested with trypsin. The resulting peptides were analyzed LC-MS/MS. The abundance of phosphopeptides containing the indicated phosphorylation site(s) were estimated from intensities reported by Proteome Discoverer and expressed as a percentage of the intensities of all phosphopeptides identified for each protein (Table S1). The indicated amino acid residues are phosphorylation sites that were confidently assigned at ≥1% of the total phosphopeptide abundance. The *inset* shows the total phosphopeptide abundance of the ΔRP mutant relative to the WT.

Based on the phenotypes observed and kinetics results, we questioned if the ΔRP mutation affects the endogenous phosphorylation of Pah1. This was examined by analyzing the electrophoretic mobility of the WT and ΔRP mutant enzymes upon SDS-PAGE using 5% polyacrylamide gels (53, 75). In a standard polyacrylamide gel, the size difference between the WT and mutant proteins made it difficult to compare the effects of phosphorylation on their electrophoretic mobility (Fig. 10*A left*). To resolve this issue, we subjected Pah1 to SDS-PAGE using a polyacrylamide gel containing the Phos-tag reagent, which traps and reduces the electrophoretic mobility of phosphorylated proteins (Fig. 10*A right*). As expected (53, 75), WT Pah1 displayed multiple diffuse bands at slower migrating positions in the gel. However, the ΔRP mutant form of Pah1 was concentrated at a lower position, suggesting that its phosphorylation is weaker than that of the WT enzyme. The difference in electrophoretic mobility is emphasized by the overlay of the densitograms of the WT and ΔRP mutant forms of Pah1 (Fig. 10*A*, right).

We sought further evidence to support the notion that the ΔRP mutation affects the endogenous phosphorylation of Pah1. Accordingly, the hyperphosphorylated WT and ΔRP mutant forms of Pah1 that were expressed and purified from *S. cerevisiae* were subject to phosphorylation site analysis by LC-MS/MS (Fig. 10*B*, Table S1). This analysis confirmed major phosphorylation sites identified for the WT protein in previous work (64). The ΔRP mutation caused reductions in the endogenous phosphorylation of Ser-511 (4.7-fold), Ser-602 (2.7-fold), Ser-744/Ser-748 (1.2-fold), and Ser-773/774 (2.2-fold) and a small increase in the phosphorylation of Ser-810/Ser-814 (Fig. 10*B*). Overall, the ΔRP mutation caused a 57% reduction in the endogenous phosphorylation of Pah1 (Fig. 10*B*, *inset*).

## Discussion

During the course of our studies to advance the underpinnings of Pah1 regulation in yeast lipid synthesis, we identified a region (*i.e.*, RP domain) within the N-terminal IDR of the protein with predicted structure (Fig. 2*B* and *C*). Initial studies indicated that this region was dispensable for Pah1 function as indicated by the ability of a truncation mutant (ΔN-NCR*b*) to complement the temperature-sensitive phenotype of the *pah1*Δ mutant (63). The studies performed here confirmed that the RP domain is dispensable for Pah1 function with respect to TAG synthesis (Fig. 4*A*), lipid droplet formation (Fig. 5*A*), and nuclear/ER membrane morphology (Fig. 6*A*). Unlike WT Pah1, Pah1-ΔRP was also functional in the absence of the Nem1-Spo7 protein phosphatase complex. The gain-of-function attributes correlated with an increase in membrane association and PAP activity. Yet, at the same time, the ΔRP mutation caused a reduction in Pah1 abundance. As discussed above, the Nem1-Spo7 complex is essential to Pah1 function; it recruits and dephosphorylates the enzyme at the nuclear/ER membrane to facilitate the dephosphorylation of PA to produce DAG (2, 46) (Fig. 1). The dephosphorylation also stimulates PAP activity (75), but renders the enzyme susceptible to 20S proteasome-mediated degradation (56) (Fig. 1).

The Pah1 attributes and phenotypes imparted by the ΔRP mutation are reminiscent of those associated with Pah1-7A (47, 48, 54). This phosphorylation-deficient mutant contains alanine residues substituted for the seven sites that are normally phosphorylated by the Pho85-Pho80 protein kinase (47, 54) (Fig. 2*A*, indicated by the ★) and most readily dephosphorylated by the Nem1-Spo7 complex (54, 75). The phosphorylation of the seven sites sequesters Pah1 in the cytosol, inhibits its PAP activity, but protects the enzyme from 20S proteasome-mediated degradation (47, 48, 54). Consequently, *nem1*Δ mutant cells expressing Pah1-7A exhibit greater membrane association of the enzyme, higher PAP activity, increased levels of TAG, and more normal nuclei when compared with the same cells expressing WT Pah1 (47, 48, 54). Given the similarities of the gain-of-function phenotypes mediated by the ΔRP and 7A mutations and that the 7A phenotypes are based on differences in the phosphorylation state of the protein led to the hypothesis that the RP domain regulates the phosphorylation of Pah1.

The electrophoretic mobility analysis upon Phos-tag SDS-PAGE of the purified WT and ΔRP mutant enzymes expressed in *S. cerevisiae* indicated that the ΔRP mutant Pah1 was deficient in its phosphorylation (Fig. 10*A*, *right*). This finding correlated with the increase in PAP catalytic efficiency (*k*_cat_/*K*_m_) of the Pah1-ΔRP when compared with the WT enzyme (Fig. 9*B*). Indeed, the dephosphorylated/unphosphorylated form of Pah1 exhibits greater PAP activity when compared with the phosphorylated form of the enzyme (47, 54, 75). Direct examination of phosphorylation status by LC/MS-MS analysis of peptides derived from the WT and ΔRP mutant proteins showed that mutation resulted in a 57% reduction in the overall phosphorylation of Pah1.

The sites most affected by ΔRP mutation included Ser-511, which is phosphorylated by casein kinase I (52); Ser-602, which is phosphorylated by Pho85-Pho80 (47), Cdc28-cyclin B (48), casein kinase I (52), and Rim11 (53); Ser-773, which is phosphorylated by protein kinases A (49) and C (50); and Ser-774, which is phosphorylated by protein kinase A (49) and casein kinase I (52) (Fig. 2*A*). Of these sites, we speculate that the 65% reduction in the phosphorylation of Ser-602, which is the major phosphorylation site in Pah1 (Fig. 10*B*) (64) and a main target of the Pho85-Pho80 protein kinase (47), is primarily responsible for the gain-of-function phenotypes imparted by the ΔRP mutation. The importance of Ser-602 in the regulation of Pah1 is emphasized by the fact that four different protein kinases phosphorylate this site (47, 48, 52, 53). The other residues that were negatively affected by the ΔRP mutation may not be as important as Ser-602 for the regulation of Pah1 as they are found in much lower abundance. It is known, however, that the phosphorylation of Pah1 at Ser-511, as mediated by casein kinase I, stimulates the subsequent phosphorylation of the enzyme by casein kinase II and inhibits subsequent phosphorylations by protein kinases A and C (52). The phosphorylations of Pah1 by these protein kinases do have an impact on Pah1 function (49, 50, 51), but not to the same extent afforded by the phosphorylation by Pho85-Pho80 (47, 48, 54). Clearly, the phosphorylation of Pah1 at multiple sites is complex and it is difficult to make definitive conclusions at this stage in the work. We also acknowledge that the RP domain itself contains two minor phosphorylation sites, namely, Thr-203 and Ser-250 (Fig. 2*A*), and we cannot rule out the possibility that loss of these sites due to the ΔRP mutation might contribute to the gain-of-function phenotypes.

Mammalian lipin proteins, which are also PAP enzymes (76, 77, 78), play essential roles in lipid metabolism and cell physiology (79, 80, 81, 82, 83, 84). Like Pah1, the PAP activity of the lipins is governed by the conserved N-LIP and HAD-like catalytic domains (38, 62). The tryptophan contained in the WRDPLVDID domain, which is essential to Pah1 function (63), is conserved in the lipin proteins as well. Lipins are also subject to phosphorylation/dephosphorylation-mediated regulation that controls their cellular location (85, 86, 87). However, there are differences between yeast Pah1 and mammalian lipins that govern their regulation. For example, the RP domain identified in this work that regulates the phosphorylation of Pah1 is not conserved in the mammalian lipins. Likewise, the C-terminal acid tail, which is required for Pah1 interaction with the Nem1-Spo7 complex at the nuclear/ER membrane (58), is not conserved in mammalian lipins. Conversely, the M-LIP domain found within the large IDR of lipin, which is important for its dimerization and membrane association (88), is not found in Pah1. Thus, while Pah1 might serve as a eukaryotic model to study the mode of action and kinetics of PAP activity, it might not serve as an ideal model to study all aspects of PAP regulation.

The RP domain is highly conserved in fungal orthologs of Pah1 (Fig. 3). These include opportunistic fungal pathogens such as *Candida albicans, Kluyveromyces lactis*, *Aspergillus fumigatus*, and *Fusarium oxysporum* (89, 90, 91). Fungal pathogens represent an under-recognized threat to public health and agriculture, and the growing frequency of anti-fungal resistant infections requires increased attention and novel strategies to combat them (92, 93, 94). That disruption in the regulation of Pah1 function through alterations in phosphorylation leads to broader disruptions in lipid synthesis and cellular growth (46) raises the suggestion that the RP domain may represent a possible therapeutic target for inhibiting growth of pathogenic fungi.

In summary, this work advances the understanding of Pah1 regulation through the identification of a novel domain that regulates the phosphorylation state of the enzyme. Future studies include confirmation of the RP domain structure and mechanism by which the RP domain controls enzyme phosphorylation.

## Experimental procedures

### Reagents

All chemicals used were reagent grade. Growth media were purchased from Difco Laboratories. Enzyme reagents for DNA manipulations were sourced from New England Biolabs. Clontech was the supplier of the carrier DNA used for yeast transformations. Millipore-Sigma was the source of silica gel TLC plates, ATP, bovine serum albumin, ampicillin, nucleotides, Triton X-100, and alkaline phosphatase-conjugated goat anti-mouse IgG antibodies (lot number: SLBG1482V; product number: A3562). Roche manufactured the protease-inhibitor cocktail tablets utilized in the study. The IgG-Sepharose, and Q-Sepharose as well as polyvinylidene difluoride membrane, and enhanced chemifluorescence substrate were acquired from GE Healthcare. Nickel-nitrilotriacetic acid agarose resin and the kits for plasmid extractions and DNA gel extractions were purchased from Qiagen. Thermo Fisher Scientific supplied the Pierce strong anion exchange columns, BODIPY 493/503, as well as the alkaline phosphatase-conjugated goat anti-rabbit IgG antibody (lot number: NJ178812; product number: 31340). Bio-Rad was the supplier of molecular mass protein standards, DNA size ladders, and reagents needed for electrophoresis and Western blotting. InstantBlue (Coomassie blue) was purchased from Expedeon. Phos-tag Acrylamide AAL-107 was acquired from Wako Chemicals. Scintillation counting supplies were purchased from National Diagnostics. All radiochemicals were purchased from PerkinElemer Life Sciences. Anti-Pah1 antibody (48) was generated in rabbits at BioSynthesis, Inc. The anti-phosphoglycerate kinase antibody (lot number: E1161; product number: 459250) was supplied by Invitrogen. Lipids were sourced from Avanti Polar Lipids.

### Strains, plasmids, and growth conditions

All plasmids used during this study are listed in Table 1. The isolation of plasmid DNA, PCR amplification, digestion, and ligation were performed with standard methods (95, 96, 97). Plasmid transformations were done as previously described for both *E. coli* (95), and *S. cerevisiae* (98). To introduce mutations in Pah1, PCR-mediated site-directed mutagenesis was employed (48). All mutations were confirmed by DNA sequencing. Plasmid pGS104 was constructed by digesting pGH315-ΔN-NCRb at the SacI and SalI sites and inserted into pGH452 at the same sites, following that 12 codons were removed by PCR-mediated site-directed deletion. Plasmid pGS108 was constructed by deleting the codons corresponding to amino acids 180 to 272 of Pah1 by PCR-mediated, site-directed deletion.

The yeast and bacterial strains that were used in this study are listed in Table 1. The growth of bacterial and yeast cultures was monitored by measuring the absorbance at 600 nm (A_600_). Yeast transformants were grown in synthetic complete (SC) media lacking appropriate nutrients for plasmid maintenance. Standard methods to culture yeast cells at 30 ^°^C were used for cell growth (95, 96). Plasmid-based expressions of Pah1, WT and its derivations, were performed in the *S. cerevisiae pah1*Δ strain SS1026 (31). To express the phosphorylated forms of Pah1, a *pah1*Δ *nem1*Δ strain SS1132 was utilized (48). To overexpress Pah1 and its derivations, the SS1132 stain harboring either pGH452 or pGS104 was inoculated into 250 ml SC-Ura (2% glucose) at A_600_ = 0.1 and incubated at 30 ^°^C for 24 h with shaking at 250 rpm. These cultures were harvested by centrifugation at 1500*g* for 10 min before being resuspended in 2 L of induction media (SC-Ura/1% raffinose/2% galactose) at an A_600_ = 0.4 and incubated at 30 ^°^C at 250 rpm until A_600_ = 1.0. Cells were harvested and processed immediately. Plasmid propagation was performed with *E. coli* strain DH5α. *E. coli* cells were grown at 37 ^°^C in lysogeny broth (LB) media (1% tryptone, 0.5% yeast extract, 1% NaCl, pH 7.0). Antibiotics (100 μg/ml ampicillin) were utilized to select for cells containing desired plasmids. For overexpression of Pah1 and its derivatives in *E. coli* cells, NiCo21(DE3)+pLysS RARE2 cells harboring pGH313 or pGS108 were grown up 1 L LB containing chloramphenicol (34 μg/ml) and ampicillin (100 μg/ml) until A_600_ = 1.0. Protein expression was induced by addition of isopropyl-β-D-thiogalactoside (IPTG) to a final concentration of 1 mM. Solid growth media contained 1.5% and 2% agar for *E. coli* and *S. cerevisiae*, respectively.

### Lipid labeling and analysis

Steady-state lipid labeling was performed in yeast with [2-^14^C]acetate as described previously (99). Cells were grown to either exponential (A_600_ ∼0.5) or stationary (A_600_ ∼3.5) phase at 30 ^°^C. Lipids were extracted following the method of Bligh and Dyer (100) and separated by one-dimensional TLC on silica-gel plates utilizing the solvent mixture hexane/diethyl ether/glacial acetic acid (40:10:1 v/v) (101). The resolved lipids were visualized by phosphorimaging with a Storm 860 Molecular Imager (GE Healthcare). Results were quantified with ImageQuant software using a standard curve of [2-^14^C]acetate.

### Fluorescence microscopy

The nuclear/ER morphology was imaged by transforming yeast cells with the SEC63-GFP plasmid (41). The percentage of cells with a round nucleus was scored from ≥4 fields of view (≥200 cells). The lipid droplets were imaged in exponential (A_600_ ∼0.5) and stationary phase cells (A_600_ ∼3.5) after a 30-min staining with 2 μM BODIPY 493/503 in phosphate-buffered saline (pH 7.4) (32). The average number of lipid droplets per cell was scored from ≥4 fields of view (≥200 cells). The microscope used to image all cells was a Nikon Eclipse Ni-U microscope with the EGFP/FITC/Cy2/AlexaFluor 488 filter and fields of view were recorded by a DS-Qi2 camera. Image analysis was performed with NIS-Elements BR software.

### Preparation of yeast cell extracts and subcellular fractionation

Yeast cell extracts were prepared from cells harvested in the exponential (A_600_ ∼0.5) and in the stationary (A_600_ ∼3.5) phases of growth. The cells were washed once with sterile water before being resuspended in 50 mM Tris-HCl (pH 7.5), 10 mM 2-mercaptoethanol, and dissolved EDTA-free protease inhibitor cocktail tablets (Roche cOmplete ULTRA Tablets). Glass beads (0.5 mm) were added and mixed with the cells before undergoing five repeats of 1-min bursts with 2-min cooling with a BioSpec Products Mini-Beadbeater-16 at 4 ^°^C. The disrupted cell slurries were centrifuged at 1500*g* for 10 min at 4 ^°^C. The supernatant (cell extract) was centrifuged at 100,000*g* for 1 h at 4 ^°^C to separate the cytosolic (supernatant) and membrane (pellet) fractions. The membrane fraction was resuspended in the same volume of disruption buffer as that of the cytosolic fraction.

### Purification of enzymes

Protein A-tagged Pah1 and its derivatives were expressed and purified from exponential phase *S. cerevisiae pah1*Δ *nem1*Δ mutant strain SS1132 by affinity chromatography with IgG-Sepharose followed by anion exchange chromatography with Q-Sepharose as described previously (64). The *pah1*Δ *nem1*Δ mutant strain lacks the Nem1-Spo7 protein phosphatase complex ensuring the hyperphosphorylation of the enzymes (54). His_6_-tagged Pah1 and its derivatives were expressed and purified from *E. coli* strain NiCo21(DE3)+pLysS RARE2 by nickel-nitrilotriacetic acid-agarose chromatography followed by anion exchange chromatography with Q-Sepharose as previously described (5, 75). The unphosphorylated form of the enzymes was ensured by their expression in *E. coli* (54). All steps were performed at 4 ^°^C. The purified enzyme preparations were stored at –80 ^°^C in 20 mM Tris-HCl (pH 8.0) containing 10% glycerol.

### PAP activity assays

PAP activity was measured for 20 min at 30 ^°^C by following the release of water-soluble ^32^P_i_ from the chloroform soluble [^32^P]PA (5000 com/nmol) (5, 102). The reaction contained 50 mM Tris-HCl (pH 7.5), 1 mM MgCl_2_, 2 mM Triton X-100, 0.2 mM PA, and purified preparations of Pah1 in a total of 100 μl. All reactions were performed in triplicate. The [^32^P]PA substrate was prepared by using DAG kinase from *E. coli* to phosphorylate DAG with [γ-^32^P]ATP (102).

### SDS-PAGE and immunoblot analysis

SDS-PAGE analyses were conducted according to standard procedures (103). To analyze the phosphorylation status of proteins *via* SDS-PAGE, Phos-tag AAL-107 (20 μM) was added to otherwise standard polyacrylamide gels. For immunoblotting, protein was transferred from polyacrylamide gel to polyvinylidene difluoride (PVDF) membranes followed by probing with rabbit anti-Pah1, mouse anti-Pgk1, and rabbit anti-Cho1 antibodies at a final concentration of 2 μg/ml. Secondary antibodies used were alkaline phosphatase-conjugated goat anti-rabbit IgG antibodies and goat anti-mouse IgG antibodies at a dilution of 1:5000. Immune complexes were detected using a chemifluorescence substrate for alkaline phosphatase. The fluorescence signals from the immunoblots were detected by Invitrogen iBright1500 imager, and the signal intensities were analyzed by the iBright Analysis Software.

### Analysis of Pah1 phosphorylation by LC-MS/MS

The endogenous phosphorylation states of the WT and ΔRP mutant forms of Pah1 (*i.e.*, expressed and purified from exponential phase *pah1*Δ *nem1*Δ cells lacking the Nem1-Spo7 protein phosphatase complex) were analyzed by LC-MS/MS at the Center for Integrative Proteomics Research at Rutgers University, NJ. The preparation and analysis were performed as previously described (64).

### Protein determination

Protein concentrations were determined by the Bradford (104) protein-dye binding assay using bovine serum albumin as the standard.

### Analyses of data

SigmaPlot software was used for statistical analysis; *p* values < 0.05 were considered as statistically significant. The enzyme kinetics module of the SigmaPlot program was used to analyze the kinetic properties of PAP activity.

## Data availability

Raw MS phosphorylation data and database search results for WT and ΔRP forms of Pah1 are deposited in the MassIVE repository (https://massive.ucsd.edu/ProteoSAFe/static/massive.jsp) with the accession number MSV000092078. All other data are contained within the manuscript or the supporting information.

## Supporting information

This article contains supporting information.

## Conflict of interest

The authors declare that they have no conflicts of interest with the contents of this article.
